# Assessing SDG indicator 6.4.2 ‘level of water stress’ at major basins level

**DOI:** 10.14324/111.444/ucloe.000026

**Published:** 2021-11-04

**Authors:** Riccardo Biancalani, Michela Marinelli

**Affiliations:** 1 FAO, viale delle Terme di Caracalla, 00153 Rome, Italy; 2 c/o FAO, viale delle Terme di Caracalla, 00153 Rome, Italy

**Keywords:** water stress, GIS, environmental flows, Sustainable Development Goals, river basin, disaggregation

## Abstract

This paper describes a method to disaggregate indicator 6.4.2 (level of water stress) by major river basins. The analysis was performed using the GlobWat soil water balance model and global geospatial data consistent with national statistics published in AQUASTAT, the FAO’s global information system on water and agriculture. When a river basin spans across more than one country, the water stress calculated by country can be very different from that calculated by the river basin as the counting of the renewable freshwater resources from one country to another is highly dependent on the official agreement and treaties that regulate the flow of those resources between countries. This problem is solved hydrologically once the accounting of the water resources is done on the major river basin as a whole. The disaggregation by the river basin allows the identification of hotspots where actions should be prioritised and reveals that the area affected by a high or critical water stress spans across all continents with the exception of Oceania. It also offers the possibility of an analysis of freshwater withdrawals by sector, which may become crucial for the definition of water management policies in the context of the economic development of a country.

## Introduction

An increasing competition for natural resources, due to climate change, urbanisation, dietary changes and industrial development, is compromising ecological integrity and agricultural productivity. Agricultural ecosystems cover nearly 40% of the terrestrial surface of the Earth [1], and few options remain globally to expand the agricultural area without significant environmental, social and economic costs [2]. Water scarcity, defined as an ‘imbalance of supply and demand’ [3], is a global problem which can affect water security even in countries with ample water resources [4]. Already 40% of the world’s rural population live in river basins that are classified as water scarce [2].

Water stress has an impact on countries of every continent and hinders the sustainability of natural resources, as well as economic and social development. By 2050, nearly 4 billion people could be subject to severe water stress [5]. Levels of water withdrawal per capita vary significantly across the world because they depend on several factors such as latitude, climate and the importance of a country’s agricultural or industrial sector. In some countries water withdrawn for irrigation only exceeds the total amount of renewable freshwater resources [2,6,7].

The Sustainable Development Goals (SDGs) aim to address these issues, and in particular SDG 6 aims to ensure the availability and sustainable management of water and sanitation for all sectors, including agriculture and the environment [8]. Target 6.4 seeks to ensure sustainable withdrawals and supply of freshwater to address water scarcity. Two indicators have been selected for monitoring the target: indicator 6.4.1 monitors the change in water-use efficiency, tracking the relation between the economic growth and the use of water resources, while indicator 6.4.2 on the level of water stress tracks how much freshwater is being withdrawn by all economic activities, compared to the total renewable freshwater resources available, after taking into account environmental flows requirements. The two indicators offer a complementary view on a country’s path to achieving target 6.4.

In order to support the policy making process towards achieving the SDGs, the monitoring system has to be capable of providing detailed and accurate information to each level of decision makers, particularly at the country and sub-country level. Methods to disaggregate the indicator at higher spatial and temporal resolutions have been already tested on a limited spatial scale [9].

In fact, while country level reports are useful for a global overview of the indicator, the Statistical Commission of the United Nations (UNSC) has stated that ‘*…improving data disaggregation is fundamental for the full implementation of the indicator framework and to fully reflect the principles of the 2030 Sustainable Development Agenda to ensure that no one is left behind, and stressed that efforts should be made to strengthen national capacities in that area and to develop the necessary statistical standards and tools’ …*’.^1^ Following this statement, the Inter-Agency and Expert Group of the Sustainable Development Goals (IAEG-SDG) established a Working Group on data disaggregation, which concluded to strongly encourage countries and custodian agencies to disaggregate the indicators following various criteria. In particular, the Working Group identified both the hydrological unit and the economic sector as the two main criteria for the disaggregation of the indicator 6.4.2 on water stress.

Disaggregating the indicator 6.4.2 will bring its expression nearer to users and stakeholders, either physically or socially and economically. That will contribute to increase the sense of participation and ownership that is needed for the ultimate achievement of the SDGs, and for ensuring that no one is left behind [10].

The aim of this paper is to present and discuss the method followed for the disaggregation of indicator 6.4.2 by major river basins and to show how the different economic sectors impact on the sustainability of water use. This fills a gap in the information provided by the previous assessment of the indicator at country level, offering a better insight to practitioners and decision-makers alike.

## Materials and methods

### Indicator 6.4.2

The development of the methodology for this indicator evolved from the existing Millennium Development Goals (MDGs) indicator 7.5: proportion of total water resources used. The MDG indicator was defined as ‘the total volume of groundwater and surface water withdrawn from their sources for human use (in the agricultural, domestic/municipal and industrial sectors), expressed as a percentage of the total actual renewable water resources’ [11].

In the preparation of the set of SDG indicators, such methodology was amended with the inclusion of the environmental flows (EF) to better reflect the condition of sustainability which characterises the SDG framework. This paper refers to the methodology for the calculation of indicator 6.4.2 described in the metadata [12] approved at the third meeting of the IAEG-SDGs at tier 1, meaning that ‘(the) indicator is conceptually clear, has an internationally established methodology and standards are available…’ [13].

Indicator 6.4.2 is calculated as the ratio between (a) the amount of total freshwater resources withdrawn and (b) the total renewable freshwater resources after subtracting the amount of water needed to support existing environmental services [12], also indicated as environmental flows. It is important to note that the total freshwater withdrawals are ‘gross’, as suggested in previous studies [14]. In other words, the indicator is calculated considering the total water abstraction and it does not consider the return flow, which is calculated as the difference between the gross water abstracted and the consumptive water use [15].

The water stress in percentage can be calculated by Equation 1:

(1) $$\text{Water Stress (\%) =}\frac{\text{Total freshwater withdrawn}}{\text{Total renewable freshwater resources }-\text{ EF}}\text{*}100$$

The purpose of this indicator is to show the degree to which water resources are being exploited to meet a country’s water demand. It measures the country’s pressure on its water resources and therefore the challenge on the sustainability of its water use. *Low water stress* indicates minimal potential impact on resource sustainability and on potential competition among users. *High water stress*, on the contrary, indicates substantial use of water resources, with greater impacts on resource sustainability and the potential for conflict among users.

The Food and Agriculture Organization of the United Nations (FAO), as the custodian agency of indicator 6.4.2, collects annual data on water stress at country level and reports the data to the Statistical Division of the United Nations (UNSD). The data collection modality is based on the use of specific questionnaires that are sent to each country every year.

**Figure 1:**
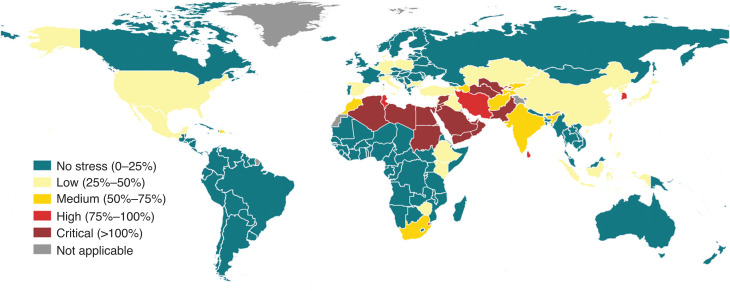
SDG 6.4.2 level of water stress (in percentage) at country level, based on the official statistical data available in AQUASTAT (reference year 2018). Geographic projection.

The questionnaires are then elaborated, including for quality control, and the resulting statistics of indicator 6.4.2 are reported in AQUASTAT [16], the FAO’s global information system on water and agriculture publicly available online. Figure 1 shows the map of the indicator at country level based on the data available for the year 2018. According to the indicator’s metadata [12], water stressed conditions occur when withdrawals exceed 25% of renewable freshwater resources. Thirty-four countries are experiencing water stress between 25% and 75%, while 25 countries are above 75% and are considered to be seriously stressed.

As said, the SDG reporting process is based on country data collected by the custodian agency. However, in the case of indicator 6.4.2 the computation by country implies the aggregation of the water resources parameters at country level with no consideration of the actual hydrography. In fact, each country may account for its water resources irrespective of how they are shared with its neighbours. This entails the possibility of a double counting of the same water resources when they flow from one country to another. Disaggregating the indicator, and recalculating it at basin level eliminates this situation, providing a different and more hydrologically sound view on the dynamics of water resources and their use.

### Disaggregation criteria

Sustainable management of water resources cannot disregard the economic needs and choices linked to their use and the environmental and demographic conditions of each area. In fact, the indicator can be calculated as the sum of the withdrawals by different economic sectors divided by the total renewable freshwater resources (TRWR), while considering the EF. This subdivision of the indicator’s equation has been implemented in order to be able to spatially distribute the aggregated data of the three parameters of Equation 1.

The economic sectors used for such purpose are those identified in the metadata of indicator SDG 6.4.1 ‘change in water use efficiency over time’ [17], in order to keep consistency between the two indicators. They are defined following the categories of the International Standard Industrial Classification of All Economic Activities (ISIC), Revision 4, as follows:

- Sector Agriculture: ISIC Section A
- Sector Industry: ISIC Sections B, C, D and F^2^
- Sector Services: ISIC Section E and Section G to T

The disaggregated formula becomes:

(2) $$\text{Water Stress (\%) =}\frac{V_{\text{A}}+V_{\text{S}}+V_{\text{M}}}{\text{TRWR}-\text{EF}}\text{*}100$$

with *V*_A_ being the volume of freshwater withdrawal by the agricultural sector, including irrigation (inclusive of nurseries), livestock (watering and cleaning) and freshwater aquaculture; *V*_S_ is the volume of freshwater withdrawal by the service sector; *V*_M_ is the volume of freshwater withdrawal by the industrial sector; TRWR is the total renewable freshwater resources and EF is the environmental flow. All the variables are expressed as volumes in million m^3^.

The data on water withdrawals in the three sectors are taken from AQUASTAT. The river basins used for this study are the 230 major river basins of the FAO World map of the major hydrological basins. This dataset was obtained by delineating drainage basin boundaries from hydrologically corrected elevation data: HydroSHEDS and Hydro1K [18]. The data on environmental flows are from the Global Environmental Flows Information System (GEFIS)^3^ database of the International Water Management Institute (IWMI) [19].

### Data and methods

The spatial disaggregation by major river basin of indicator 6.4.2 was implemented for the three main economic sectors. Withdrawal data available in AQUASTAT for the year 2018 have been spatialised using proxies or related variables as explained in the following sections.

#### Total renewable freshwater resources

TRWR refer to the freshwater available for use in a territory and include surface water (lakes, rivers and streams) and groundwater. In this paper the TRWR at basin level have been estimated through GlobWat [20], a global water balance model used by the FAO to assess water use in irrigated agriculture. GlobWat can be downloaded online, and it is based on spatially distributed high-resolution datasets that are consistent at a global level and calibrated against long-term averages for internal renewable water resources, as published in AQUASTAT.

GlobWat calculates the water balance in two steps: 1) a ‘vertical’ water balance is calculated per pixel, it includes evaporation from in situ rainfall (‘green’ water) and incremental evaporation from irrigated crops; 2) a ‘horizontal’ water balance is calculated by each basin to determine discharges from river (sub-)basins, taking into account incremental evaporation from irrigation, open water and wetlands (‘blue’ water). The results of the water balance calculations consist of monthly values by grid cell for generated precipitation, actual evaporation, incremental evaporation due to irrigated agriculture, surface runoff,^4^ groundwater recharge and water stored as soil moisture.

To assess the TRWR of each major river basin annually, we have considered the sum of the annual drainage and of the annual groundwater recharge estimated by the model by basin:

(3) $$\text{TRWR = P}-{\text{ET}}_{\text{act}}\text{=Drainage + GW}$$

with P being the precipitation, ET_act_ is the actual evapotranspiration (water consumption), Drainage is the surface runoff (million m^3^), and GW is the groundwater recharge (million m^3^).

#### Environmental flows

In the computation of indicator 6.4.2, environmental flows are ‘…*the quantity and timing of freshwater flows and levels necessary to sustain aquatic ecosystems which, in turn, support human cultures, economies, sustainable livelihoods, and wellbeing*’ [21]. Water quality and the resulting ecosystem services are excluded from this formulation, which is confined to water volumes. This does not imply that water quality and the support to societies, which are dependent on environmental flows, are not critical issues that should not be taken care of. They are indeed addressed by other targets and indicators of the SDG system, such as 6.3.1 (Proportion of domestic and industrial wastewater flow safely treated), 6.3.2 (Proportion of bodies of water with good ambient water quality), 6.5.1 [Degree of integrated water resources management implementation (0–100)] and 6.6.1 (Change in the extent of water-related ecosystems over time).

**Figure 2:**
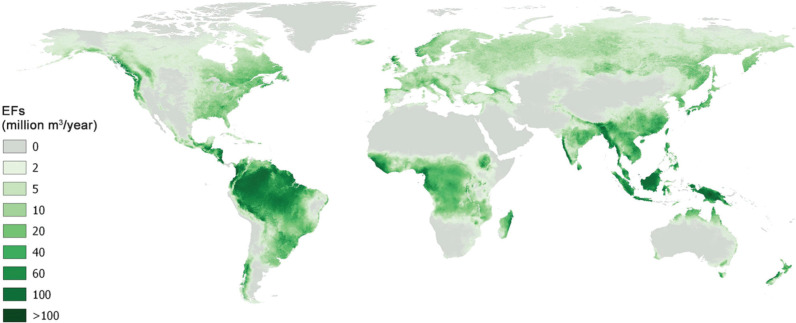
Environmental flows (EF) based on the values calculated in GEFIS (http://gef.iwmi.org/) and adjusted to the total renewable water resources estimated with GlobWat. Geographic projection. Resolution 5 arc-minutes (approximately 10 km at the equator).

In this work, EFs were assessed using the data published online by the IWMI in the GEFIS, which provides the value of EF as a percentage of the total actual flow. Such a percentage value has been then applied to the amount of TRWR as estimated by GlobWat, in order to have a volume of EF which is consistent with the estimation of water resources available in AQUASTAT. The map of the EF volumes is shown in Fig. 2.

#### Total freshwater withdrawal

Total freshwater withdrawal (TFWW) is defined as the sum of the relevant withdrawals in the three main economic sectors of agriculture, industry and services.

TFWW includes freshwater and fossil groundwater. It does not include direct use of non-conventional water, such as direct use of treated wastewater, direct use of agricultural drainage water and desalinated water. In AQUASTAT total water withdrawals by sector include the non-conventional water sources. For this reason, to be consistent with the equation of indicator 6.4.2, TFWW was calculated as expressed in Equation 4:

(4) $$\text{TFWW = }\sum {\text{ww}}_{\text{e}}–\sum {\text{du}}_{\text{n}}$$

with TFWW being the total freshwater withdrawal (million m^3^); ww_e_ is the water withdrawal (million m^3^) for the economic sector ‘e’ (agriculture; industry; services); du_n_ is the direct water use (million m^3^) from a non-conventional source ‘n’ (direct use of wastewater; direct use of agricultural drainage water; use of desalinated water).

Data on the amount of non-conventional water resources are rare and scattered in AQUASTAT. However, when available, for this paper it was assumed that the drainage water and treated wastewater are mainly used for irrigation and that the desalinated water is mostly used for domestic purposes.^5^

**Figure 3:**
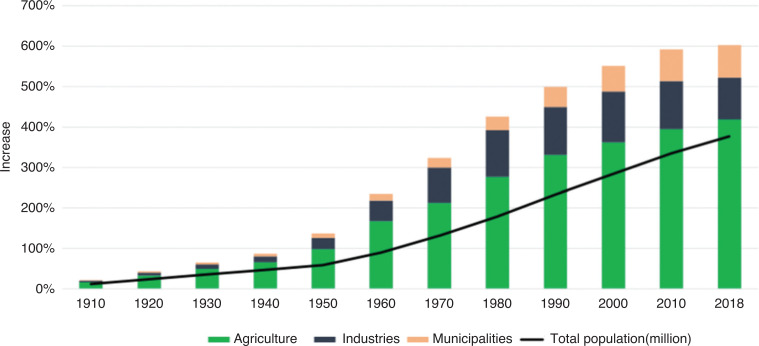
Percentage increase (respect to year 1900) of total population and water withdrawal (km^3^ by sector) in the last century. Global water withdrawal and world population from 1900 to 2018. Source: Shiklomanov 2000 [22] for water withdrawal 1990–2000; AQUASTAT for 2010 and 2018. Population data from FAOSTAT [23].

In 2018, at a global level, the withdrawal ratios were 72% agriculture, 12% services^6^ and 16% industry [16]. While agriculture still has a major share of water withdrawals, in the last decades the rate of increase of water withdrawal in the other two sectors has been proportionally much faster (Fig. 3).

Of the three main factors driving the increase of water withdrawals (population growth, economic development and change in consumption patterns), population seems to be particularly relevant, as domestic demand will rise by more than threefold in all African and Asian subregions, and it will more than double in Central and South America [24]. By combining the global water withdrawal with the world population, it is possible to notice that the world population increased almost 4 times over the last century while water withdrawals increased 6 times over the same period.

The following sections will describe the approaches used to geo-spatialise the freshwater withdrawal in each sector.

#### Agriculture

The agriculture freshwater withdrawal (*V*_A_) is the volume of water withdrawn for the agricultural sector, including for irrigation (inclusive of nurseries), livestock (watering and cleaning) and freshwater aquaculture.

Unfortunately, data disaggregated for irrigation, livestock and aquaculture are available only for a few countries. When available, however, irrigation water withdrawal ranges between 70% and 90% of the overall agriculture water withdrawal [16]. Therefore, irrigation water withdrawal has been taken as a proxy to estimate *V*_A_.

**Figure 4:**
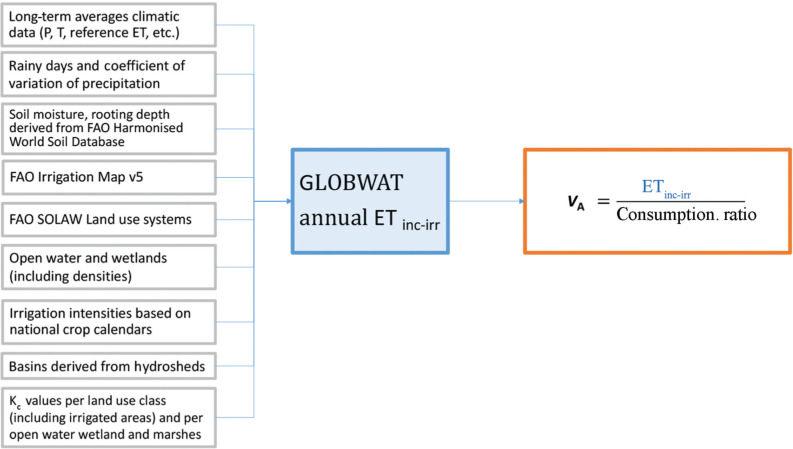
Approach used to spatialise the agriculture freshwater withdrawal (*V*_A_).

**Figure 5:**
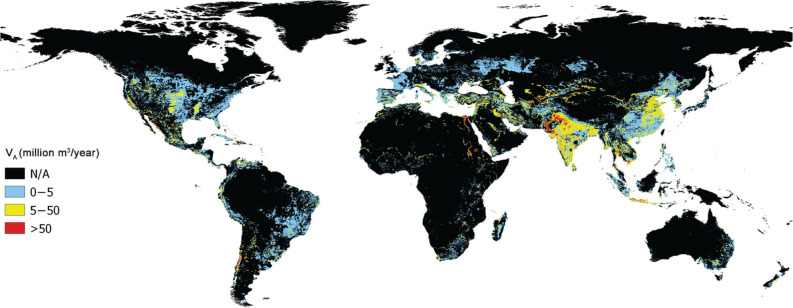
Agriculture freshwater withdrawal for the year 2018 (*V*_A 2018_) in million m^3^. Geographic projection. Resolution 5 arc-minutes (approximately 10 km at the equator).

To assess the volume of water withdrawn for agriculture, we used GlobWat to assess the annual incremental evapotranspiration due to irrigation (ET_inc-irr_). This is an estimation of the irrigation water *consumed* in irrigated areas, that is, the share of the water withdrawn actually used by the crop or evaporated from the ground. From ET_inc-irr_, the spatialisation was derived through the consumptive ratio, defined as the ratio between 1) ET_inc-irr_ estimated with GlobWat and 2) *V*_A_ for the year 2018 available in AQUASTAT (Fig. 4). Figure 5 shows the global map of *V*_A_ for the year 2018.

#### Services

The services freshwater withdrawal (*V*_S_) is the volume of water withdrawn for the service sector. In AQUASTAT the sectors included in ‘services’ are referred to as ‘municipal’. It is usually computed as the total freshwater withdrawn by the public distribution networks.

**Figure 6:**
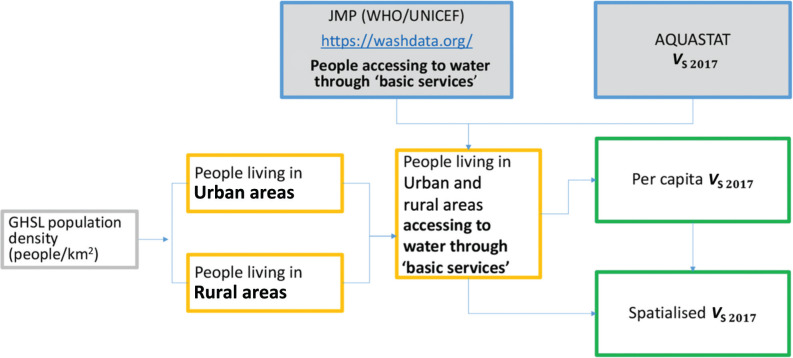
Approach used to spatialise the service freshwater withdrawal (*V*_S_).

**Figure A1:**
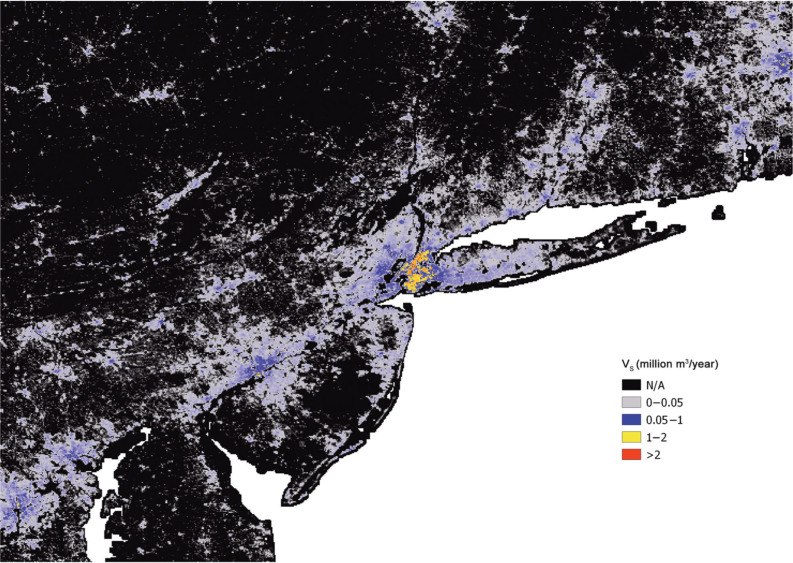
Service water withdrawal for the year 2018 (*V*_s 2018_) spatialised using the GHSL population density layer and the JMP database (access to water through ‘basic services’). This figure shows the New York area in the United States. Geographic projection. Spatial resolution 30 arc-seconds (approximately 1 km at the equator).

The volume of water withdrawn by the service or municipal sector largely depends on the number of people living in a certain area. Therefore, for this sector we started from the analysis of the population density [25] and then we considered the access to water through ‘basic services’ both in rural and urban areas. This category includes all the people who can access water through an infrastructure or through a walking distance less than 30 minutes (Fig. 6). Then, using the data available in AQUASTAT, the service water withdrawal per capita was calculated for each country and finally the spatialised global map of the service water withdrawal (*V*_S 2018_) was drawn (Fig. A1). The dataset used for the population is the Global Human Settlement Layer for the year 2015 (GHSL-2015) [26], which also provides a useful classification of the populated places in rural and urban areas, according to predefined density thresholds. The GHSL-2015 has been adjusted to the year 2018 by multiplying it by the ratio between the national population of each country in the years 2018 and 2015. To determine the number of people accessing to water through ‘basic services’, we used the dataset produced by the Joint Monitoring Programme (JMP) [27] for Water Supply, Sanitation and Hygiene. For those countries for which JMP data were not available, the analysis was based only on the Global Human Settlement Layer (GHSL) population data (e.g., Timor Leste).

#### Industry

The industry freshwater withdrawal (*V*_M_) is the volume of water withdrawn for mining and quarrying, manufacturing, construction and energy. This sector refers to self-supplied industries not connected to the public distribution network. It includes water for the cooling of thermoelectric and nuclear power plants, but it does not include hydropower.

Globally, approximately 16% of total water withdrawals are used for industrial purposes. Industrial water use has the largest share in high-income countries with a total of 48% in Europe and North America [16].

Considering that global data on the distribution of industrial settlements are not available, it was assumed that the population density layer (GHSL) [26], based on the Nighttime Lights satellite data, would provide a good proxy of where electricity is requested and consumed and thus where industries are located throughout the world, in order to estimate how much water each inhabitant uses for this sector.

**Figure 7:**
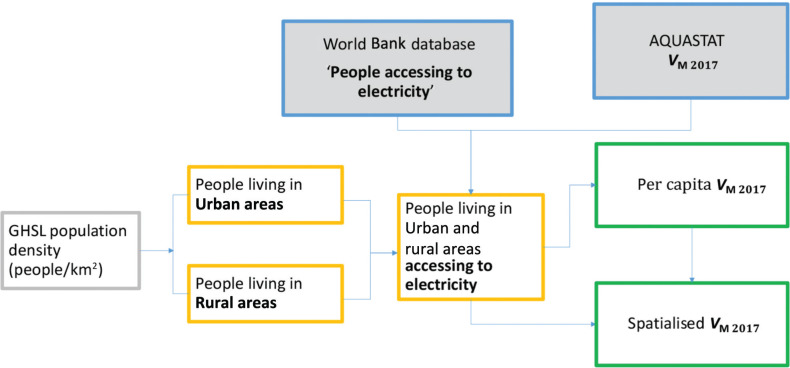
Approach used to spatialise the industry freshwater withdrawal (*V*_M_).

As shown in Fig. 7, it was decided to apply an approach analogous to the one used for the spatialsation of *V*_S_.

**Figure A2:**
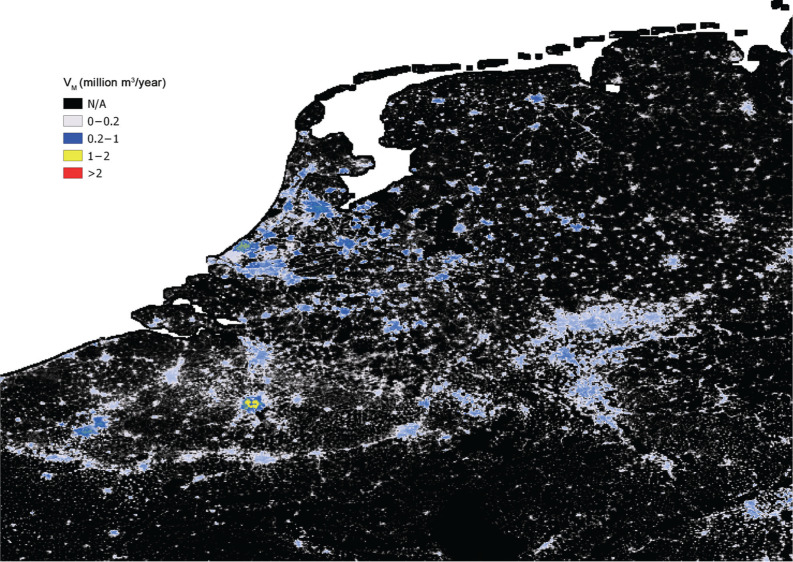
Industry water withdrawal for the year 2018 (*V*_M_ 2018) spatialised using the GHSL population density layer and the World Bank database (‘access to electricity’). This figure shows an area of the Netherlands and Northern Germany. Geographic projection. Spatial resolution 30 arc-seconds (approximately 1 km at the equator).

Starting from the population density, we considered the percentage of people with *access to electricity* and living in rural and urban areas. This information has been publicly available for several years on the World Bank website [28]. Then, using AQUASTAT data, we calculated the industrial freshwater withdrawal per inhabitant per year and finally the global map industrial freshwater withdrawal (*V*_M 2018_) expressed in volumes (Fig. A2).

This analysis suffers of some weak points that will be discussed in the limitation section of this paper. Here, we wish to note that the assumption that the nightlights can correctly identify the areas of production and consumption of electricity for industrial purposes would need to be revised as new data become available.

## Results and discussion

**Figure 8:**
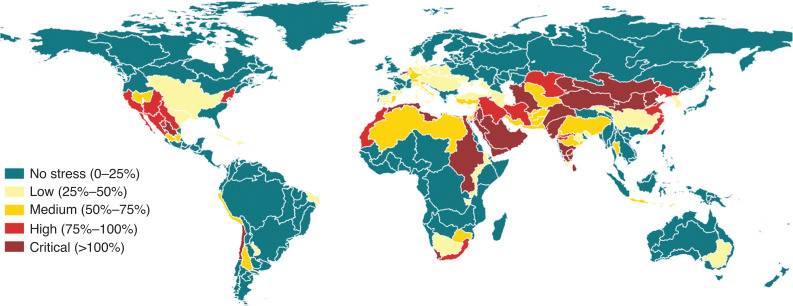
SDG 6.4.2 – Level of water stress, by major river basin expressed in percentages. Reference year 2018. Geographic projection.

The calculation of the SDG 6.4.2 by basin was carried out using the FAO global map of hydrological basins, derived from Hydrosheds and downloadable from Geonetwork, the FAO geospatial catalogue (http://www.fao.org/geonetwork). By aggregating all the variables described in the previous sections by major basins, the resulting SDG 6.4.2 map is shown in Fig. 8.

The analysis of country data on water stress showed that countries that may appear to have a low level of water stress can be part of a much more stressed basin. In fact, when a river basin spans across more than one country, the water stress calculated by country can be very different from the one computed at the river basin level due to the double counting of the renewable freshwater resources from one country to another. This problem is solved once the accounting of the water resources is done on the major river basin as a whole. Following the thresholds established in the metadata for this indicator [12], major river basins with an indicator’s level lower than 25% have no water stress. Those basins with a water stress greater than 75% have a high or critical water stress. High values of water stress mean more water users are competing for limited water supplies.

Compared with the map of water stress at country level (Fig. 1), the disaggregation by river basin reveals that the area affected by a severe water stress spans across all continents with the exception of Oceania. This is not evident from the map of the indicator at country level and may have relevant implications for the formulation of appropriate water management policies in the interested areas. Disaggregating the indicator offers another perspective, which may become particularly important in the context of the economic development of a country and the consequent changes in the structure of its economy.

**Figure A3:**
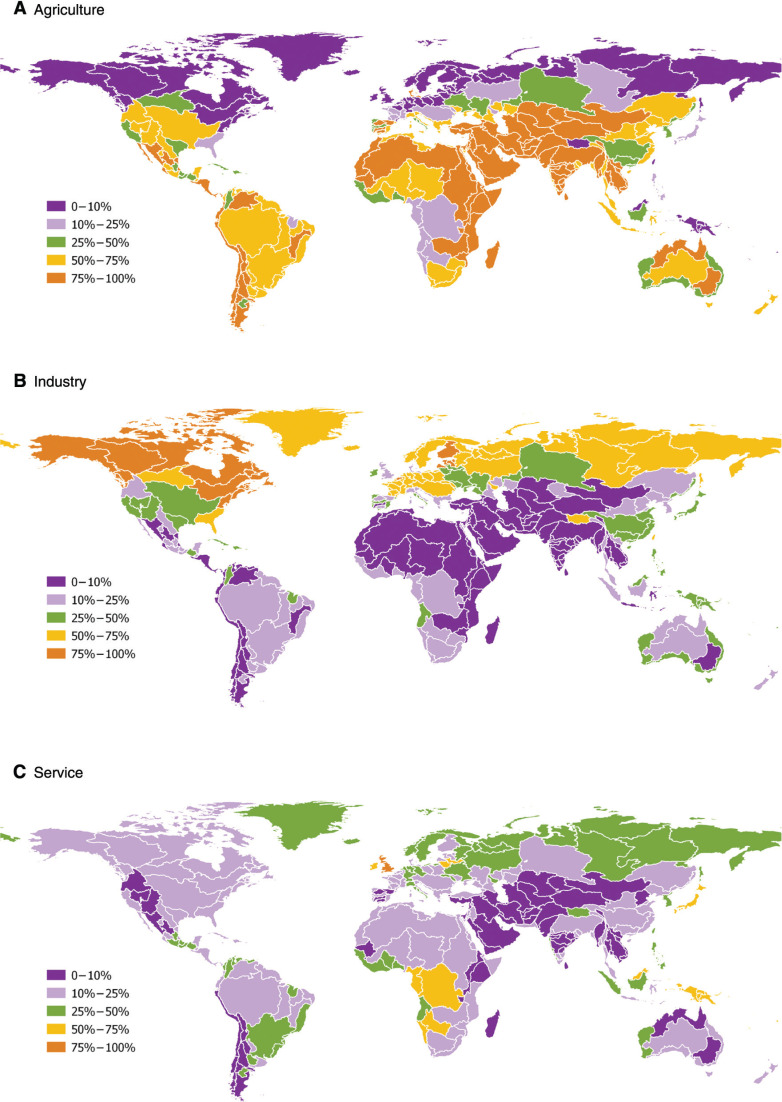
Proportion of the agriculture (A), industry (B), and service (C) sector freshwater withdrawal with respect to the total freshwater withdrawal per major river basin (reference year 2018). Geographic projection.

**Figure A4:**
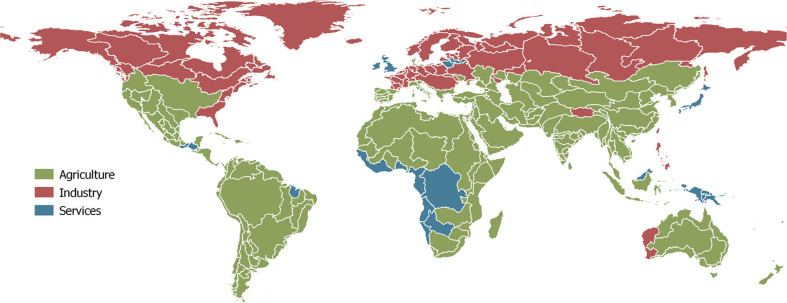
Dominant sector freshwater withdrawal per major river basin (reference year 2018). Geographic projection.

**Figure A5:**
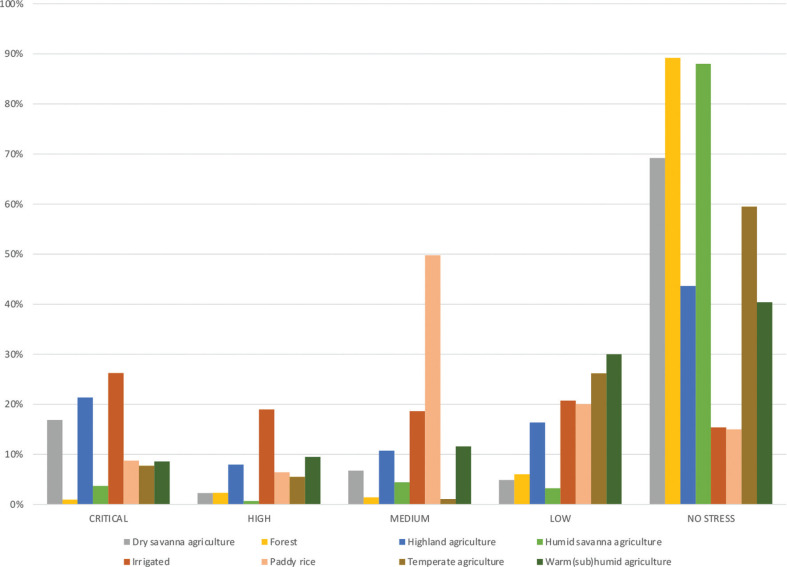
Analysis of the occurrence of some of the major agricultural systems in the classes of water stress.

The possibility of analysing the indicator and its components against other spatially distributed information (e.g., population density, land cover, precipitation, etc.) allows increasing the value of the information provided by the indicator alone. As agriculture is the main water user (Figs A3 and A4), we have analysed the major agricultural systems [29] against the classes of water stress. Irrigated agriculture is the most frequent type of agricultural system in basins with high and critical water stress while paddy rice is prevalent in medium stressed basins (Fig. A5). Enabling conditions to optimise water use by increasing the crop water productivity is essential for these areas.

One of the objections related to this water stress indicator is that it does not consider the return flow, which could be a relevant component in some countries, as has also been demonstrated by recent studies [30]. Vanham et al. [31], elaborate on this point, coming to the suggestion to calculate two versions of the indicator, with and without the computation of the return flow. While the metadata of the SDG indicator cannot be modified unilaterally, its interpretation can be improved and facilitated by providing such information.

Statistical data on the return flow are not available in most cases. However, we have considered water consumption to be a good proxy for the difference between the water withdrawal and the return flow. Consequently, by replacing freshwater withdrawal with water consumption in the indicator formula, we can have an idea of the impact of the return flow on water stress:

(5) $${\mathrm{WS}}_{\text{c}}\text{ (\%) =}\frac{{\mathrm{ET}}_{\text{inc-irr}}+{\mathrm{(V}}_{\text{S}}\text{* 0.1)}+{\mathrm{(V}}_{\text{M}}\text{* 0.1)}}{\text{TRWR}-\text{EF}}\text{*}100$$

with WS_c_ being the water stress calculated considering water consumption; ET_inc-irr_ is the incremental ET due to irrigation (derived from GlobWat); *V*_S_ is the service freshwater withdrawal, *V*_M_ is the industrial freshwater withdrawal, TRWR is the total renewable freshwater resources (derived from GlobWat) and EF is the environmental flows (based on GEFIS). All these variables are expressed in volumes (million m^3^).

For estimating the consumptive use, a return flow of about 90% was assumed for the services and industrial sectors. For the agricultural sector, the incremental actual ET due to irrigation (estimated using GlobWat) was used as a proxy.

**Figure 9:**
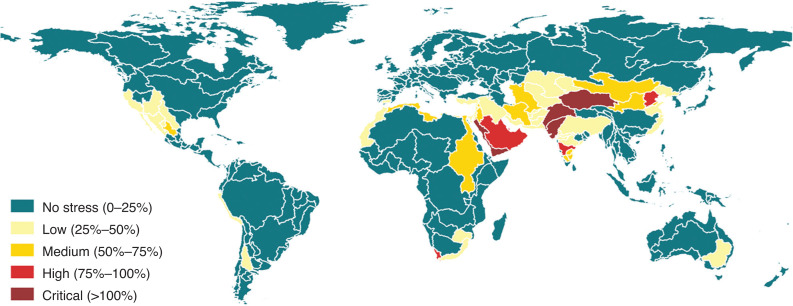
Water stress indicator calculated using the water consumption (percentages). Reference year 2018. Geographic projection.

Following these considerations, the same method used to disaggregate Equation 2 has been applied to Equation 5, so as to verify the feasibility of the approach proposed by Vanham et al. [31]. The result is a disaggregated map of the indicator calculated on the basis of water consumption instead of water withdrawal, at the major basin level (Fig. 9).

The maps in Figs 8 and 9 show an overall picture of the pressure exerted by all kinds of human activity on water resources, allowing an analysis based on the natural hydrology and the spatial distribution of those pressures.

## Limitations

One of major efforts of this study was to ensure a consistency between the AQUASTAT national statistical data available for each economic sector and the global geospatial datasets used for their spatialisation. For example, for the service and industrial sectors, the GHSL database for the year 2015 [26] was harmonised to the reference year of the study, using the national population data for the year 2018 available in AQUASTAT [16].

In addition, in the absence of a global layer of the industrialised areas, it was assumed that the population density layer (GHSL) [26], based on the Nighttime Lights satellite data, would provide a good proxy of where electricity is requested and consumed and thus where industries are located throughout the world, in order to estimate how much water each inhabitant uses for this sector.

As mentioned above, the lack of consistent datasets at a global level for each of the variables needed to compute the indicator is a cause of concern about the robustness of the results. The aim of this paper is to present the distribution of water stress at major river basins level. That has been done by using the existing global datasets, supported using proxies when necessary, as described in the Methods section. However, we recognise that stronger efforts should be made to improve the data collection at the country level, to contribute to the reliability of the global studies as well as providing a stronger basis for the computation of the indicator at the country level.

About the uncertainty due to the model, it was mitigated by calibrating the GlobWat against the values for internal renewable water resources as published in AQUASTAT, and its validation was done against mean annual river basin outflows. However, it is worth considering that not all the input data of the GlobWat model are consistent with the reference year of the study, for example, the irrigation density map [32] refers to the year 2013. Moreover, the model does not take into account the inter-basin water transfer, which is a limitation of most hydrological models.

To summarise, the proposed method presents limitations due to both the availability of robust data and the hydrological model. To overcome those limitations, it is necessary to promote the collection and processing of data on water resources and water use by countries, to be used to create a harmonised global set of data. More accurate datasets will also allow better calibration of the hydrological models. Moreover, models could be improved by implementing the possibility of accounting for the water inter-basin transfers.

In light of these challenges, we will continue our research on the disaggregation with the objective of improving the quality of the results once more accurate and recent global datasets become available for this topic.

## Conclusions

The disaggregation of the water stress indicator by major basins highlights the importance of the proper consideration of the hydrological conditions when assessing the pressure that the use of water for human needs puts on natural water resources. This gives a more comprehensive view of the global distribution of water stress, increasing the granularity of the information and allowing the identification of those cases where country level assessments may be hiding situations that might be relevant for implementing an integrated management of water resources at the regional or sub-regional level.

Such analysis also provides the basis for bringing the disaggregation exercise at the sub-basin level, so as to supply decision makers with more articulated information on the availability of water resources within a country.

Disaggregating the indicator offers the possibility of an analysis of freshwater withdrawals by sector, which may become particularly important for the definition of water management policies in the context of the economic development of a country and the resulting changes in the structure of its economy.

Finally, considering the role of water consumption offers a further insight into the detail of the dynamics of water use. Such information, properly combined with the spatial disaggregation, would provide essential data to plan a more sustainable use of water resources, particularly in water scarce basins and countries.

## Data Availability

The datasets generated during and/or analysed during the current study are available from the corresponding author on reasonable request.

## Notes

1 See Decision 47/101/n from the 47th UN Statistical Commission (https://unstats.un.org/unsd/statcom/47th-session/documents/Report-on-the-47th-session-of-the-statistical-commission-E.pdf).

2 The industry sector is called ‘MIMEC’ in the metadata of indicator 6.4.1. It includes mining and quarrying, manufacturing, construction and energy.

3 Global Environmental Flows Information System (http://eflows.iwmi.org/).

4 Surface runoff is termed ‘drainage’ in GlobWat.

5 For Oman, Qatar and Cabo Verde the desalinated water is greater than the water withdrawal for services, so it was assumed that the surplus is used for the industrial sector. This implies that for these countries the freshwater withdrawal for services is considered ‘0’.

6 In AQUASTAT services are termed as ‘municipal’.

## Abbreviations

FAO: Food and Agriculture Organization of the United Nations
GEFIS: Global Environmental Flow Information System
GHSL: Global Human Settlement Layer
IAEG-SDGs: Inter-Agency and Expert Group of the Sustainable Development Goals
IISD: International Institute for Sustainable Development
ISIC: International Standard Industrial Classification of All Economic Activities
IWMI: International Water Management Institute
JMP: Joint Monitoring Programme

## References

[1] Ramankutty N, Evan TA, Monfreda C, Foley AJ (2008). Farming the planet: 1. Geographic distribution of global agricultural lands in the year 2000. Global Biogeochemical Cycles.

[2] FAO (2017). The future of food and agriculture – trends and challenges.

[3] FAO (2012). Coping with water scarcity. An action framework for agriculture and food security.

[4] Ahopelto L, Veijalainen N, Guillaume JHA, Marko Keskinen M, Marttunen M, Varis O (2019). Can there be water scarcity with abundance of water? Analyzing water stress during a severe drought in Finland. Sustainability.

[5] Sadoff CW, Hall JW, Grey D, Aerts JCJH, Ait-Kadi M, Brown C (2015). Securing water, sustaining growth.

[6] Scheierling S, Treguer DO, Booker JF (2016). Water productivity in agriculture: looking for water in the agricultural productivity and efficiency literature. Water Econ Policy (WEP).

[7] WWAP (United Nations World Water Assessment Programme) (2016). The United Nations World Water Development Report 2016: water and jobs.

[8] United Nations (2015). Transforming our world: the 2030 Agenda for Sustainable Development.

[9] Fehri R, Khlifi S, Vanclooster M (2019). Disaggregating SDG-6 water stress indicator at different spatial and temporal scales in Tunisia. Sci Total Environ.

[10] IISD (2017). Disaggregated data is essential to leave no one behind.

[11] UNSD (2000). Indicator 7.5: Proportion of total water resources used.

[12] UNSTATS (2020). Metadata of indicator 6.4.2.

[13] IAEG-SDGs (2016). Tier classification for global SDG indicators.

[14] Smakhtin VU, Revenga C, Doll P (2004). Taking into account environmental water requirements in global-scale water resources assessments.

[15] SEEA. Central framework glossary (2012). https://seea.un.org/sites/seea.un.org/files/documents/seea_glossary_terms_languages_v2.pdf.

[16] FAO (2021). AQUASTAT: FAO’s global information system on water and agriculture.

[17] UNSTATS (2019). Metadata of indicator 6.4.1.

[18] FAO (2011). Hydrological basins derived from Hydroshed.

[19] Sood A, Smakhtin V, Eriyagama N, Villholth KG, Liyanage N, Wada Y (2017). Global environmental flow information for the sustainable development goals.

[20] Hoogeveen J, Faurès JM, Peiser L, Burke J, Van De Giesen N (2015). GlobWat – a global water balance model to assess water use in irrigated agriculture. Hydrol Earth Syst Sci.

[21] Dickens C, Smakhtin V, Biancalani R, Villholth KG, Eriyagama N, Marinelli M (2019). Guidelines for a minimum standard method for global reporting.

[22] Shiklomanov IA (2000). Appraisal and assessment of world water resources. Water Int.

[23] FAO FAOSTAT Food and Agriculture Organization of the United Nations Corporate Statistical Database.

[24] Burek P, Satoh Y, Fischer G, Kahil MT, Scherzer A, Tramberend S (2016). Water Futures and Solution: Fast Track Initiative (Final Report). IIASA Working Paper WP-16-006.

[25] Florczyk AJ, Corbane C, Ehrlich D, Freire S, Kemper T, Maffenini L (2019). GHSL data package 2019.

[26] Schiavina M, Freire K, MacManus K (2019). European Commission, Joint Research Centre (JRC).

[27] Joint Monitoring Programme for Water Supply, Sanitation and Hygiene (2019). Led by the World Health Organization and United Nations Children’s Fund (WHO/UNICEF).

[28] SE4ALL (2010). World Bank, Sustainable Energy for All database, from the SE4ALL Global Tracking Framework led jointly by the World Bank, International Energy Agency, and the Energy Sector Management Assistance Program.

[29] FAO (2011). The state of the world’s land and water resources for food and agriculture (SOLAW). Managing systems at risk.

[30] Simons GWH, Bastiaanssen WGM, Cheema MJM, Ahmad B, Immerzeel WW (2020). A novel method to quantify consumed fractions and non-consumptive use of irrigation water: application to the Indus Basin Irrigation System of Pakistan. Agric Water Manag.

[31] Vanham D, Hoekstra AY, Wada Y, Bouraoui F, de Roo A, Mekonnen MM (2018). Physical water scarcity metrics for monitoring progress towards SDG target 6.4: an evaluation of indicator 6.4.2 ‘Level of water stress’. Sci Total Environ.

[32] Siebert S, Henrich V, Frenken K, Burke J (2013). Update of the digital global map of irrigation areas to version 5.

